# An Efficient Strategy for Heterologous Expression and Purification of Active Peptide Hainantoxin-IV

**DOI:** 10.1371/journal.pone.0117099

**Published:** 2015-02-03

**Authors:** Hui Zhang, Peng-Fei Huang, Er Meng, Wen-Ying Li, Lu Zhou, Ling-Yun Zhu, Lei Wu, Meng-Jie Li, Song-Ping Liang, Dong-Yi Zhang

**Affiliations:** 1 Key Laboratory of Protein Chemistry and Developmental Biology of the Ministry of Education, College of Life Sciences, Hunan Normal University, Changsha, Hunan 410081, China; 2 Research Center of Biological Information, College of Science, National University of Defense Technology, Changsha, Hunan 410073, China; University of New South Wales, AUSTRALIA

## Abstract

Hainantoxin-IV (HNTX-IV) from the venom of the spider *Selenocosmia hainana* is a potent antagonist that specifically inhibits the tetrodotoxin-sensitive (TTX-S) sodium channels. The toxin peptide consists of 35 amino acids and adopts a typical inhibitory cystine knot (ICK) motif. To obtain adequate HNTX-IV peptides for further insight into the structure-activity relationships of the toxin, a novel strategy including cloning, expression and purification was developed in an *E. coli* expression system. For this purpose, a seamless restriction-free (RF) cloning method was employed for the construction of an expression vector to avoid introducing unwanted sequences into the target gene. Furthermore, the solubility of recombinant HNTX-IV could be promoted efficiently by the combination of a glutathione S-transferase (GST) tag and a small ubiquitin-related modifier (SUMO) tag. Finally, an affinity-chromatography-free purification strategy was developed by cut-off dialysis tubing combined with trichloroacetic acid (TCA) extraction. Further HPLC purification yielded recombinant, tag-free HNTX-IV with high yield and purity. The molecular weight of recombinant HNTX-IV (rHNTX-IV) is identical to its theoretical value according to Matrix-Assisted Laser Desorption / Ionization Time of Flight Mass Spectrometry (MALDI-TOF-MS) analysis. The recombinant toxin has similar activity (IC_50_ value of 120 nM) on the tetrodotoxin-sensitive (TTX-S) sodium channels in adult rat dorsal root ganglion (DRG) neurons to native toxins. In the report, an efficient and cost-effective strategy for producing rHNTX-IV was developed, which paved the way for the further study of structure-activity relationships of rHNTX-IV and its pharmaceutical applications.

## Introduction

HNTX-IV is a 35 amino-acid neuronal toxin with an inhibitory cystine knot (ICK) motif isolated from the venom of the Chinese bird spider *Selenocosmia hainana* [1]. The sequence of HNTX-IV is ECLGFGKGCNPSNDQCCKSSNLVCSRKHRWCKYEI-NH_2_. The toxin can inhibit TTX-S voltage-gated sodium channels with an IC_50_ value of 34.0 nM in adult rat dorsal root ganglion (DRG) neurons. Because of its compact ICK motif and robust activity, HNTX-IV can act as a potential scaffold for drug design. HNTX-IV was traditionally obtained through natural sources [2] or solid-phase peptide synthesis [3]. However, its low abundance in venom produces a low yield, and the correct folding of disulfide-rich peptides by chemical synthesis is always time-consuming and difficult.

To obtain adequate HNTX-IV for the further study of structure-activity relationships and exploration of its pharmaceutical applications by molecular engineering, an efficient, simple and cost-effective strategy for the functional production of HNTX-IV in *E. coli* was established in this report. In our study, GST (glutathione S-transferase) combined with SUMO was utilized to promote the solubility and folding of rHNTX-IV. The GST fusion tag is widely used to promote the solubility and folding of recombinant proteins [4,5]. SUMO, as a novel tag, has gained popularity [6–9] for its powerful solubilization capacity as well as its robust and specific removal by SUMO protease, leaving a native sequence of toxin peptides [10]. The functional expression of rHNTX-IV in BL21(DE3) was accomplished by the combination of GST and SUMO without additional refolding processes in our study.

Furthermore, an affinity-chromatography-independent purification strategy was developed by introducing TCA precipitation to purify rHNTX-IV peptides. Another improvement is that a ligation-independent RF (restriction free) cloning method [11] was adopted to construct the rHNTX-IV expression vector, so there are no additional residues added to the rHNTX-IV peptide. Most importantly, this recombinant HNTX-IV had similar biological activity to natural HNTX-IV. In conclusion, we provided a simple and efficient strategy for the recombinant expression and purification of bioactive rHNTX-IV.

## Materials and Methods

### Ethics Statement

All animal experimental procedures were approved by the Ethical and Animal Welfare Committee of Hunan Normal University.

### Materials

*E. coli* DH5α competent cells were purchased from TaKaRa (Otsu, Shiga, Japan). The BL21(DE3) strain, Rosetta strain, and pET-43a (+) vector were purchased from Novagen (Madison, WI, USA). The SHuffle strain was from NEB (Beverly, MA, USA). The cDNA of HNTX-IV [12] and GST-tag were kept in our laboratory. The DNA polymerase KOD-plus-Neo was purchased from TOYOBO (Osaka，Japan). Primer synthesis, DNA sequencing and SUMO-tag synthesis were performed by Sangon (Shanghai, China). All of the chemicals and reagents were purchased from Sigma (St. Louis, MO, USA). Sprague-Dawley rats were purchased from the Xiangya animal room of Central South University. SDS-PAGE gels NuPAGE and Seeblue Plus2 pre-stained molecular weight marker were purchased from Invitrogen (Carlsbad, CA, USA).

### Construction of pET-GS-HNTX-IV vector

**Fig 1:**
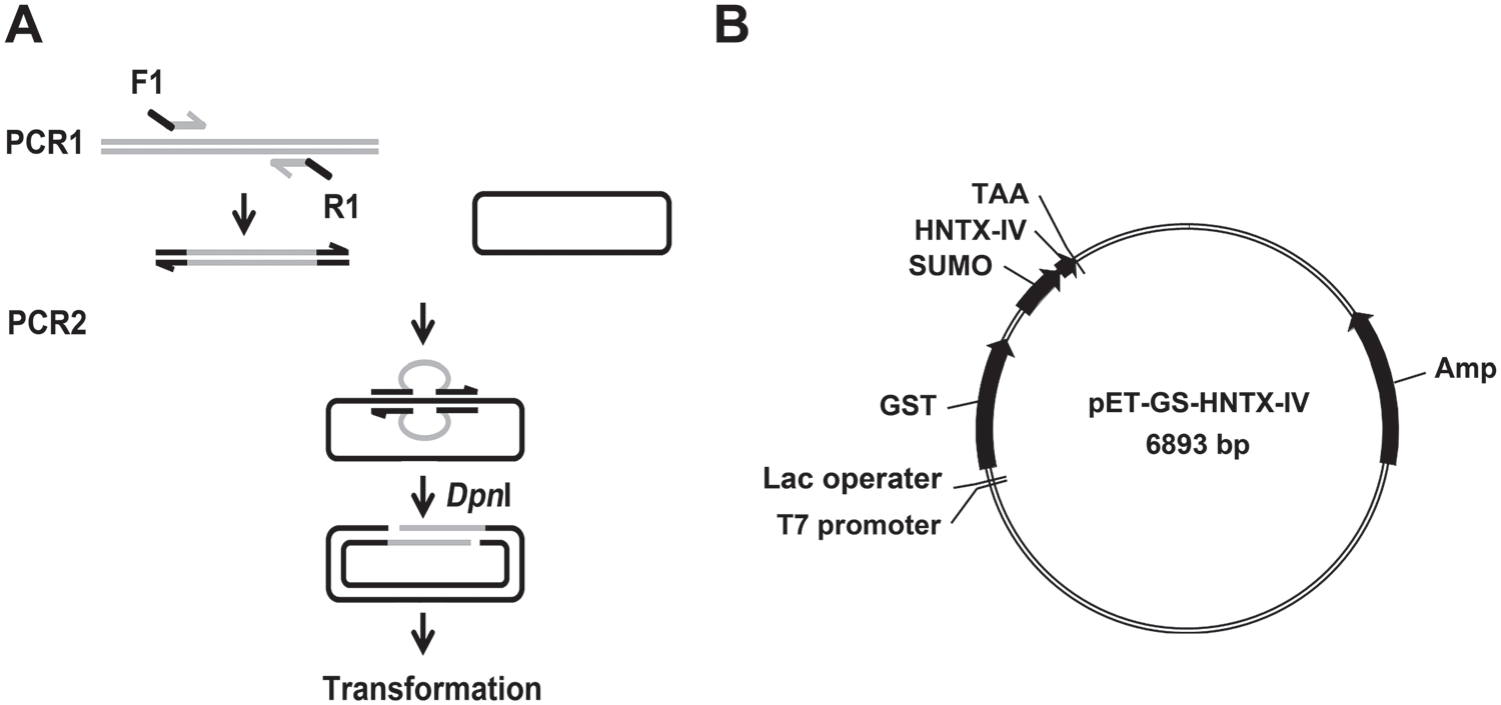
Construction of pET-GS-HNTX-IV expression vector. A) Schematic diagram of RF-cloning strategy. F1: forward primer. R1: reverse primer. The portion complementary with the target gene is marked gray, and the portion complementary to the recipient vector is marked black. B) Map of pET-GS-HNTX-IV expression vector.

The RF cloning strategy was applied to construct the expression vector (Fig. 1A). The GST sequence was integrated into the backbone of pET-43a in place of the NusA tag by the following primer pair: 5ʹ-GAAATAATTTTGTTTAACTTTAAGAAGGAGATATACAT *ATGTCCCCTATACTAGGTTATTG*-3ʹ (forward primer); 5ʹ-GATGGTGATGGTGATGACCAGAACCACTAGTAGTCACGATGCG GCCGCTC-3ʹ (reverse primer). The underlined sequences of the forward primer and the reverse primer were complementary to the 5ʹ end and the 3ʹ end of the insertion or replacement point of the recipient vector pET-43a (+) with a *T*_*m*_ value of 80°C, respectively. The italicized portions were complementary to the 5ʹ end and the 3ʹ end of GST with *T*_*m*_ value of 60°C, respectively. The resultant construct was verified by sequencing and named pET-GST. Similarly, the SUMO sequence was cloned into the pET-GST vector downstream of GST by the primer pair: 5ʹ-TCCGGGAGCTCGTGGATCCGAATTCTCTGACTCTGAAGTTAACCAGGAAG-3ʹ (Sumo—forward); 5ʹ-CGATGGTACCGTCGACGTCCTGCAG *ACCACCGATCTGTTCACGGTGAG*-3ʹ (Sumo-reverse). The construct was verified by sequencing and named pET-GS. Subsequently, the HNTX-IV gene with a stop codon was inserted behind the SUMO sequence by the primer pair: 5ʹ-TCGAAGCTCACCGTGAACAGATCGGTGGTGAGTGCTTAGGGTTTGG-3ʹ (HNTX-IV-forward); 5ʹ-CGATGGTACCGTCGACGTCCTGCAGTTATATTTCATATTTACACCACC-3ʹ (HNTX-IV-reverse). The resulting plasmid was named pET-GS-HNTX-IV. The map of the expression vector is shown in Fig. 1B.

### Expression of recombinant protein

The pET-GS-HNTX-IV plasmid was chemically transformed into *E. coli* BL21(DE3) competent cells. After incubating overnight, one colony from the LB plate was picked and inoculated into 5 ml LB medium with 100 μg/ml ampicillin sodium, which was incubated at 37°C in a shaker at 220 rpm until the OD_600_ reached 0.6. The 5 ml seed culture was transferred into 500 ml LB medium supplemented with 100 μg/ml ampicillin sodium, which was further incubated at 37°C in a shaker at 220 rpm until the OD_600_ reached 0.6. The culture was induced by 0.2 mM IPTG at 220 rpm and 37°C for 4 h. The induced bacteria were harvested by centrifugation at 4500 × g for 10 min. The pellet was suspended using 20 ml PBS buffer (38.7 mM Na_2_HPO_4_, 11.3 mM NaH_2_PO_4_, 150 mM NaCl, pH adjusted to 7.4) complemented with 1 mM PMSF, 5% glycerol, 1 mM MgCl_2_, 10 mg/L RNaseA and 20 mg/L DNase I. Cells were lysed by sonication for 5 min at 20% amplitude using a Branson SONIFIER S-250D (Emerson, USA) in ice water. The lysates were centrifuged at 15000 × g and 4°C for 30 min. The supernatant was stored at 4°C for further purification. Induction at 16°C was carried out overnight to verify the optimal temperature for rHNTX-IV production.

### Purification of GST-SUMO-HNTX-IV fusion protein by GST affinity chromatography

The supernatant was loaded onto a 5 ml pre-equilibrated glutathione resin column with a flow rate of 0.5 ml/min. Flow-through was applied to the column once again to ensure maximal binding. The resin was washed by 5 column volumes with Ca^2+^- and Mg^2+^-free PBS. The GST-tagged protein was eluted by 2 resin-bed volumes of elution buffer (10 mM Tris-HCl, 1 mM EDTA, 150 mM NaCl, 200 mM glutathione, pH 8.0).

### Preparation of recombinant SUMO protease and removal of the GST-SUMO tag by SUMO protease

SUMO protease was produced in *E. coli*, purified as described previously, and stored in 50% glycerol in -20°C [8,10]. 30 μg recombinant SUMO protease was applied to digest 1 mg GST-SUMO-rHNTX-IV at 30°C for 1 h or at 4°C overnight.

### TCA, acetonitrile and acetone precipitation

TCA, acetonitrile and acetone were tested for the removal of tags and intact recombinant protein after SUMO protease cleavage individually. For TCA precipitation, 1/10 protein volume of 100% TCA was added into the digested protein solution, kept at 4°C for 10 min, and then centrifuged for 15 min at 4°C and 15000 × g. For acetone precipitation, 4 volumes of cold acetone were added and kept at -20°C for 10 min then centrifuged for 15 min at 4°C and 15000 × g. Acetonitrile precipitation was performed as previously described [13]: the cleaved tag and unspecific proteins were precipitated by acetonitrile in the ratio of 2:1 (acetonitrile: buffer: v/v) and kept at -20°C for 10 min. The soluble fractions containing the peptides were separated by centrifugation at 15000 × g for 20 min at 4°C. The supernatant and precipitates were collected for SDS-PAGE analysis. The supernatant was dialyzed against water by 1 kDa dialysis tubing and lyophilized for HPLC purification.

### Affinity-chromatography-independent purification of rHNTX-IV

After sonication and centrifugation, instead of chromatography affinity, the supernatant of the induced cell lysates was subject to dialysis by 20 kDa cut-off dialysis tubing against Tris-HCl buffer (50 mM Tris-HCl, pH 8.0, 150 mM NaCl) overnight. Subsequently, the dialyzed solution was digested by SUMO protease at 30°C for 1 h or 4°C overnight. Then, the cleaved peptides were extracted by TCA precipitation, acetonitrile precipitation or acetone precipitation. The supernatant was dialyzed against distilled water by 1 kDa dialysis tubing with three buffer changes and lyophilized for HPLC purification.

### Purification of rHNTX-IV by RP-HPLC

The dialyzed solution was lyophilized and dissolved in 500 μl distilled water and filtered through 0.45 μm micro-filter before loading. Then, the solution was applied to a Symmetry C18 (4.6 × 250 mm, 5 μm) column and eluted at a flow rate of 1 ml/min using a gradient of 5%–45% buffer B (0.1% TFA in acetonitrile) over 35 min after washing with buffer A (0.1% TFA in ddH_2_O) for 15 min to clear residual salts or small molecules. The eluted compounds were detected by UV absorption at 215 nm. The eluted peaks were collected and lyophilized then stored at -20°C.

### Identification of rHNTX-IV by mass spectrometry

Molecular mass identification of rHNTX-IV was performed on a Voyager-DE^TM^ STR MALDI-TOF mass spectrometer (Applied Biosystems, Voyager-DE STR Biospectometry workstation). Ionization was achieved by irradiation with a nitrogen laser (337 nm), with a 20-kV acceleration voltage. α-Cyano-4-hydroxy-cinnamic acid was used as the matrix.

### Culture of dorsal root ganglion neurons (DRGs)

Sprague-Dawley rats (30 days old) of both sexes were euthanized by CO_2_ asphyxiation and decapitated. DRG neurons were harvested from rats and collected in Dulbecco’s modified Eagle’s medium. The DRGs were treated with 20 U/ml protease for 20 min followed by 0.28 U/ml collagenase for 40 min. Neurons were dissociated in Ham’s F12 medium supplemented with 10% horse serum, 100 U/ml penicillin, 100 μg/ml streptomycin, and 3.0 mM L-glutamine. Cells were plated on glass coverslips coated with poly-L-lysine and maintained at 37°C in a 95% O_2_, 5% CO_2_ incubator for 24 h before electrophysiological recordings [14].

### Whole cell patch-clamp experiments

Ionic currents were recorded under the whole cell patch-clamp mode using an EPC-10 amplifier and the Pulse program (HEKA Electronics). Patch pipettes fabricated from borosilicate glass tubes using a P-97 puller (Sutter Instruments) were pulled to a resistance of 2.0–2.9 MΩ after heat-polishing. The membrane currents were usually filtered at 5 kHz and sampled at 20 kHz. Voltage errors were minimized using 60%–80% series-resistance compensation, and the capacitance artifact was canceled using the amplifier circuitry. Because the leak currents were relatively small and could be a nonlinear function of the voltage, no electronic compensation for the voltage-dependent leak current was used [15].

On DRG neurons, for recording TTX-sensitive sodium currents, the pipette solution contained 145 mM CsCl, 4 mM MgCl_2_, 10 mM HEPES, 10 mM EGTA, 10 mM glucose, and 2 mM ATP (pH 7.2); and the bath solution contained 145 mM NaCl, 2.5 mM KCl, 1.5 mM CaCl_2_, 1.2 mM MgCl_2_, 10 mM HEPES, and 10 mM glucose (pH 7.4). Tetrodotoxin (TTX, 300 nM) was added to the bath solution when recording the TTX-Resistant currents from the DRG neurons. For recording calcium currents, the internal solution contained 110 mM Cs-methane sulfonate, 14 mM phosphocreatine, 10 mM HEPES, 10 mM EGTA, and 5 mM ATP-Mg (pH 7.3); and the external solution contained 10 mM BaCl_2_, 125 mM tetraethylammonium-Cl, 0.3 mM TTX and 10 mM HEPES (pH 7.4). To eliminate any influence of differences in osmotic pressure, all of the internal and external solutions were adjusted to 280±5 mOsmol/L with sucrose.

Electrophysiological data were analyzed and displayed by Pulsefit (HEKA Electronics). Curve fitting and statistical analysis were performed with OriginPro 9.0 (OriginLab) and GraphPad Prism 5.0 software. The statistical significance was determined at *p*<0.05 and calculated with the two-tailed Student’s t test. The data are presented as the mean ± SEM.

## Results

### Expression of GST-SUMO-HNTX-IV in BL21(DE3)

**Fig 2:**
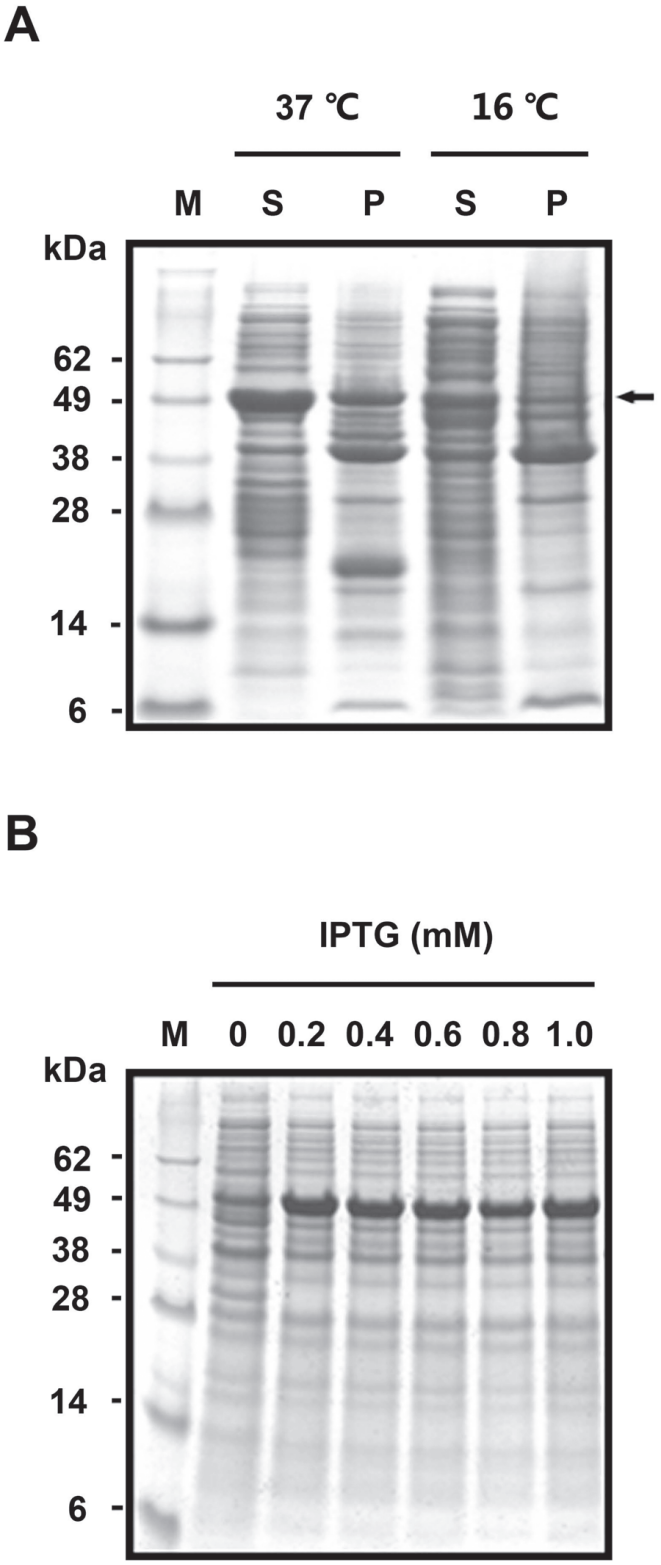
Expression of GST-SUMO tagged HNTX-IV in BL21(DE3). A) Solubility analysis at 37°C and 16°C. M: protein molecular weight marker; S: soluble fraction. P: precipitates. The target protein is denoted by an arrow. B) The expression level under different IPTG conditions.

The BL21(DE3) strain was selected as the expression host because of its rapid growth rate, with the OD_600_ reaching 0.6 in approximately 3 h; compared with Rosetta (DE3), approximately 4 h; and SHuffle, more than 5 h (data not shown). Parameters including temperature and IPTG concentration were optimized to improve the target protein solubility and yield. Inductions at 37°C for 4 h and 16°C overnight were compared. As shown in Fig. 2A, at 37°C, the target proteins existed both in the soluble form and as inclusion bodies. The solubility was improved at 16°C, whereas the resultant protein yield decreased. Furthermore, the concentration of IPTG ranging from 0.2 mM to 1.0 mM had no obvious effect on the yield of target proteins (Fig. 2B). In our study, induction at 37°C for 4 h by 0.2 mM IPTG was chosen as the expression condition for rHNTX-IV.

### Affinity-chromatography-independent purification of rHNTX-IV

**Fig 3:**
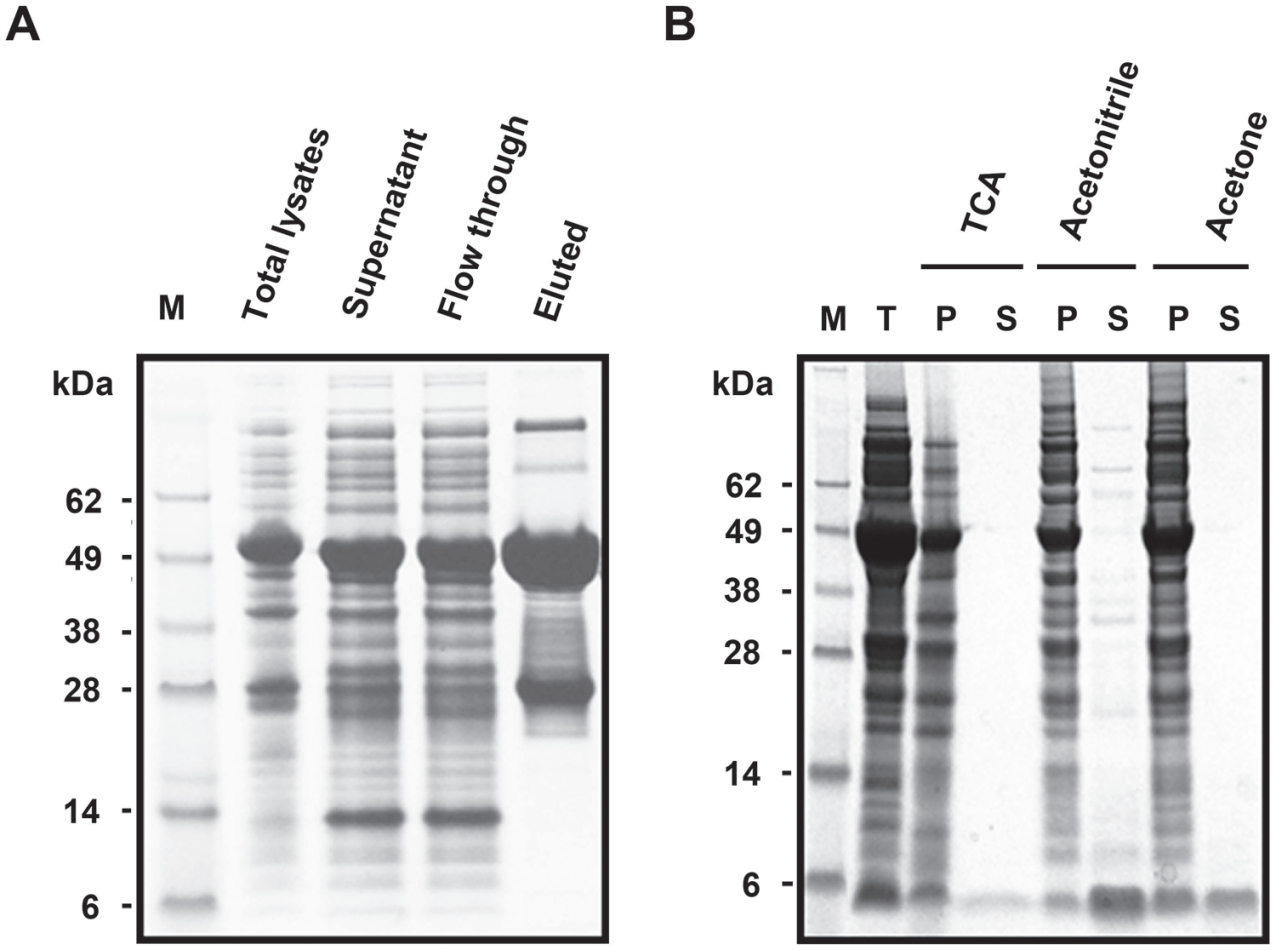
Purification of rHNTX-IV. A) Purification by glutathione column. B) Affinity chromatography independent purification by TCA, acetonitrile and acetone. T: the total cell extract digested by SUMO protease; P: the precipitated fraction; S: the soluble fraction.

The recombinant GST-SUMO-rHNTX was routinely purified by GST affinity chromatography. However, the recovery efficiency was less than 10%, even though excessive glutathione resin was used (Fig. 3A).

**Fig 4:**
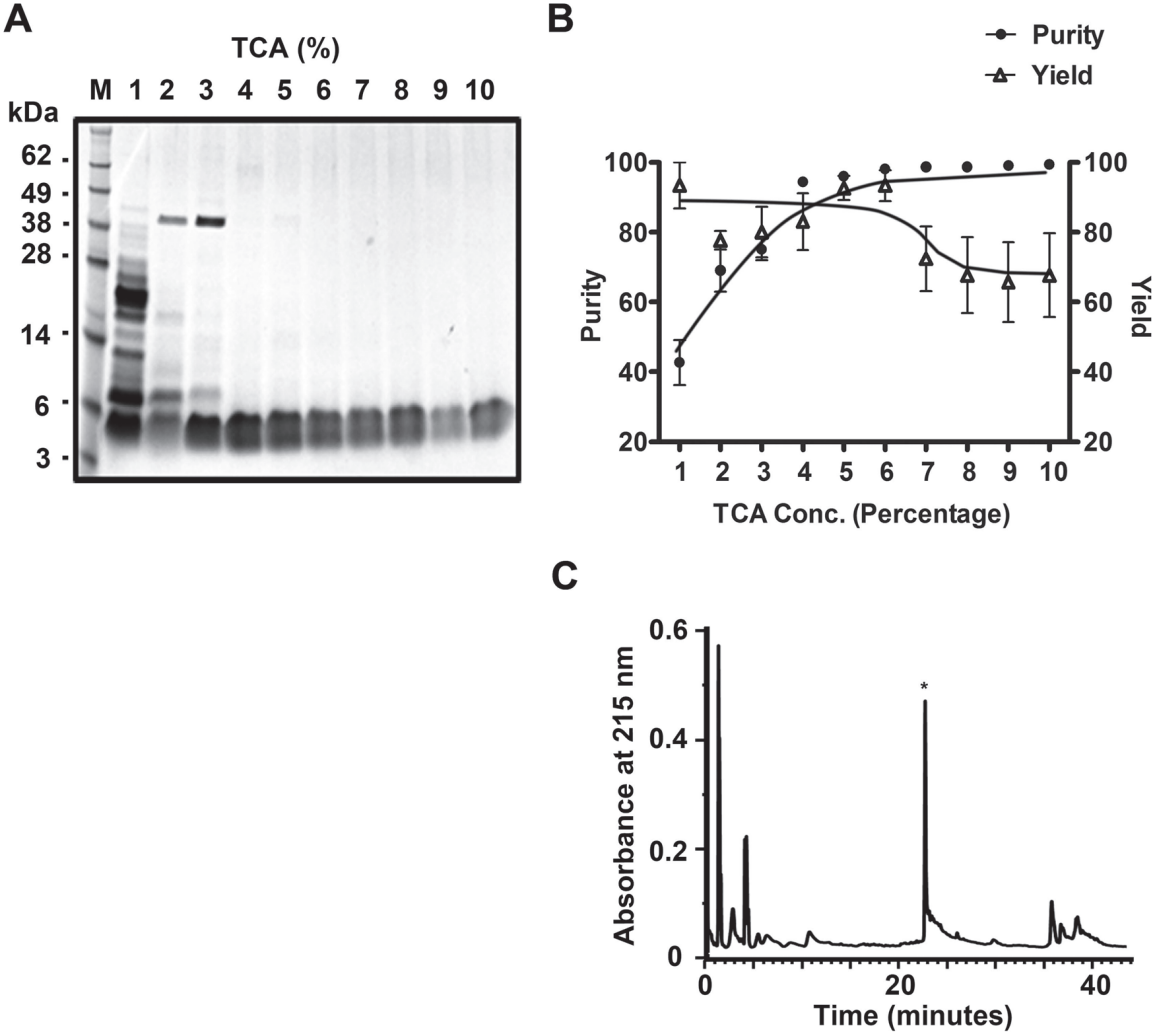
Affinity chromatography independent purification of rHNTX-IV by TCA. A) M: Protein molecular weight marker; 1–10: fractions extracted by TCA from 1% to 10%; P: precipitates. B) Relative purity (compared with lane 10) and relative yield (compared with lane 1) of rHNTX-IV under different TCA concentrations. The value was calculated by grayscale by Quantity One software. The data points (means ± S.E.) come from three independent experiments. C) Purification by RP-HPLC.

With respect to the low affinity of GST-SUMO-rHNTX-IV for the glutathione beads, GST-SUMO-rHNTX-IV in the crude cell extract was digested directly by SUMO protease without affinity purification. Given the robustness of the recombinant enzyme prepared in-house in various buffers and volumes (data not shown), the dialyzed lysates (20 ml) were completely digested by 300 μg SUMO protease at 4°C overnight. Because there were dramatic differences in hydrophobicity between the GST-SUMO tagged protein and rHNTX-IV, TCA precipitation (10% TCA), acetonitrile precipitation and acetone precipitation were tested for the removal of the GST-SUMO tag and unspecific proteins. The results showed that TCA precipitation and acetone precipitation can retrieve target peptides with high purity, while acetonitrile is less efficient (Fig. 3B). After TCA precipitation or acetone precipitation, the only band detected was between 3 kDa and 6 kDa, which was in accordance with the predicted size of rHNTX-IV. Among these methods, TCA precipitation is more suitable for the large-scale production of rHNTX-IV because less TCA is required. Because a high concentration of TCA might have a harmful effect on the peptides, a gradient of the TCA concentration ranging from 1% to 10% was tested for precipitation efficiency. 4% TCA could also clear protein contaminants efficiently (Fig. 4A, 4B). The bands between 3 kDa to 6 kDa tended to smear. This might be a result of overloading and the band could get more specific if less sample were loaded as shown in Fig. 3B. They were taken as a single band for greyscale analysis using Quantity One software, and it’s user manual can be freely downloaded from http://www.bio-rad.com/webroot/web/pdf/lsr/literature/10002940.pdf.

**Fig 5:**
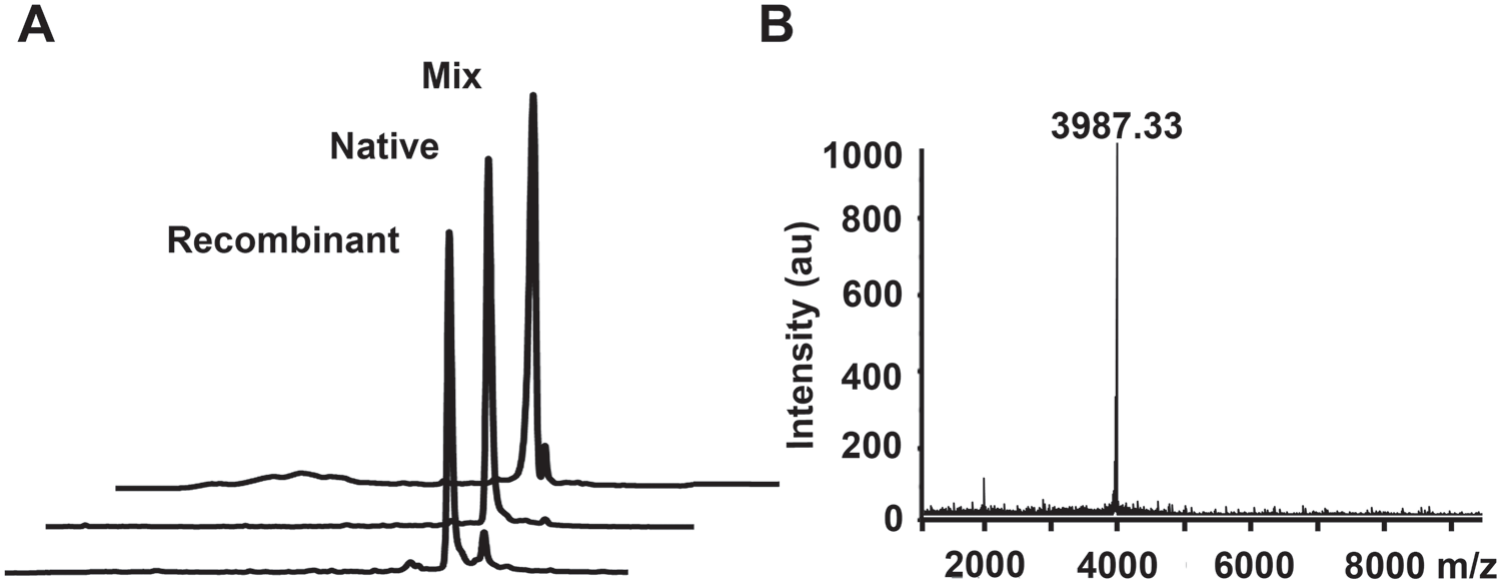
Identification of rHNTX-IV. A) Comparison of rHNTX-IV and native HNTX-IV on RP-HPLC. B) MALDI-TOF analysis of rHNTX-IV.

The peptide supernatant after TCA precipitation could be easily purified by RP-HPLC (Fig. 4C). The final yield per liter of pure rHNTX-IV was above 2 mg, compared with that of rHNTX-IV purified by GST affinity chromatography (approximately 0.2 mg per litter) as determined by Bradford assay. Furthermore, the purified rHNTX-IV was further compared with the native toxin by analytical RP-HPLC. rHNTX-IV and native HNTX-IV were eluted at the same retention time (Fig. 5A), partially proving their identical conformation [9]. The molecular weight of the eluted fraction was further identified by mass spectrometry and determined to be 3987.33 Da, which is identical to the theoretical value of HNTX-IV (Fig. 5B).

### Identification and characterization of rHNTX-IV on DRG

**Fig 6:**
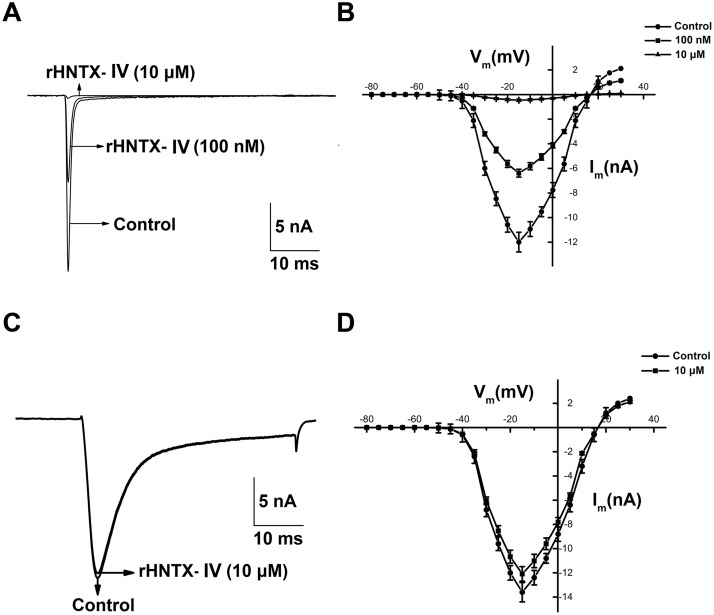
Functional characterization of the venom on voltage-gated sodium channels. A) Typical current traces from TTX-S Na^+^ channel before and after application of 100 nM and 10 μM rHNTX- IV. B) The I-V curve of TTX-S *I*_Na_ with 100 nM and 10 μM rHNTX- IV. C) Typical current traces from TTX-R Na^+^ channel before and after application of 10 μM rHNTX- IV. D) The I-V curve of TTX-R *I*_Na_ with 100 nM rHNTX- IV. The data points (means ± S.E.) come from at least five cells.

**Fig 7:**
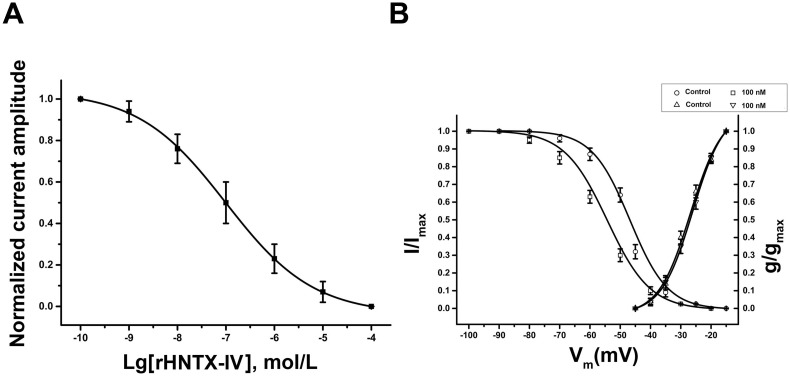
The kinetics curves of rHNTX-IV. A) The dose-effects curve of rHNTX-IV. The data points (means ± S.E.) are fit to the Hill equation. B) The steady-state inactivation and activation curves for TTX-S *I*_Na_ with 100 nM rHNTX-IV. The data points (means ± S.E.) come from at least five cells and are fit to the Boltzmann equation.

**Fig 8:**
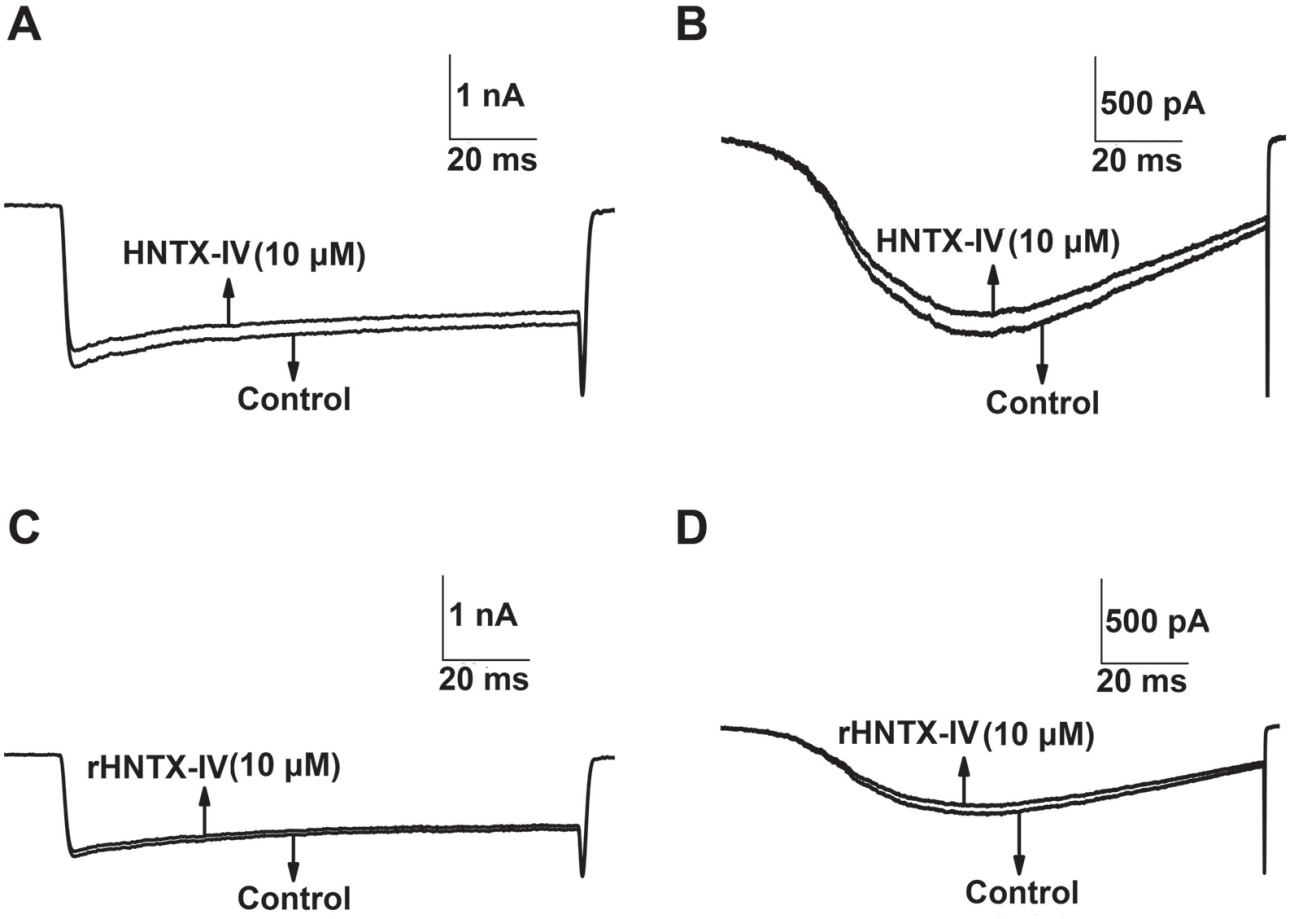
Functional characterization of the venom on voltage-gated calcium channels. A) Typical current traces from an L-type Ca^2+^ channel before and after application of 10 μM native HNTX-IV; B. Typical current traces from a T-type Ca^2+^ channel before and after application of 10 μM native HNTX- IV. C) Typical current traces from an L-type Ca^2+^ channel before and after application of 10 μM rHNTX- IV. D) Typical current traces from a T-type Ca^2+^ channel before and after application of 10 μM rHNTX-IV.

In our study, 100 nM rHNTX-IV inhibited approximately 40% TTX-S *I*_Na_, and 10 μM rHNTX-IV inhibited TTX-S *I*_Na_ currents by more than 90% (Fig. 6A). The currents induced before the toxin application were used as controls. The IC_50_ value was 0.12 ± 0.02 μM (Fig. 7A), similar to that of native HNTX-IV (34 nM) [2]. Although the native HNTX-IV is amidated, it’s obvious that amidation does not affect the activity of HNTX-IV as strongly as HWTX-IV [9]. At the same concentration (10 μM), rHNTX-IV had no noticeable effects on the TTX-R *I*_Na_ (Fig. 6C), and the I-V curves of both TTX-S and TTX-R *I*_Na_ display no shift at all (Fig. 6B, 6D). In the kinetic studies, 100 nM rHNTX-IV shifted the V_0.5_ of the inactivation curve by 10 mV from -46.01 ± 0.72 to -55.27 ± 0.92 mV (n = 5), indicating that the inactivation of the sodium channel was enhanced in the presence of rHNTX-IV. On the other hand, 100 nM rHNTX-IV showed little influence on the activation curve (V_0.5_ from −26.02 ± 0.51 to −27.34 ± 0.22 mV) (n = 5) (Fig. 7B). With the electrophysiological studies, the results demonstrated similar inhibitory activity of rHNTX-IV and native HNTX-IV (IC_50_ = 34 nM). Beyond that, additional experiments were performed to support the results. It was found neither native HNTX-IV (Fig. 8A, 8B) nor rHNTX-IV (Fig. 8C, 8D) could cause obvious changes to the Ca^2+^ currents in DRGs.

## Discussion

Spider venoms represent a rich source for biologically active compounds, such as ion channel modulators [16], bioinsecticides [17], and antimicrobial peptides [18]. There are more than 42,700 extant species of spiders, and more than 10 million bioactive peptides [19] are likely to be present in the venoms of spiders, holding considerable value for potential pharmacological and physiological applications. However, only 0.01% of the peptides have been characterized, and their characterization, to a large extent, is hampered by the limited amount of available spider toxins [20,21].

One plausible approach to solve the problem is recombinant expression in *E. coli* because of its high growth rate, low cost and labor efficiency [22]. However, the folding of recombinant disulfide-rich peptides in *E. coli* is not easy [19, 22]. The folding conditions and yield for each peptides are varied and peptide-specific.

In our study, we found that fusion tags could be essential for the functional expression of disulfide bond-rich toxins. Additionally, the temperature has an obvious impact on the solubility of recombinant proteins. Because toxin peptides tend to aggregate into inclusion bodies, fusion tags such as NusA, GST, thioredoxin and SUMO are useful for solubility. However, improved solubility might not guarantee the proper folding of CKTs even if combined with the folding-assisted tags (DsbC, TrxA) [23]. The screening of folding-assisted tags for a certain toxin is still a trial and error process.

In our study, by a combination of GST and SUMO, rHNTX-IV, a potent antagonist that acts at site 1 on tetrodotoxin-sensitive (TTX-S) sodium channels with 3 pairs of disulfide bonds, was functionally expressed in BL21(DE3). As is traditionally known, the reducing environment of the cytoplasm of BL21(DE3) is not preferable for disulfide-bond formation. However, as suggested by the identical molecular weight of rHNTX-IV and its theoretical value, and a similar activity with the native toxin, the disulfide bonds of rHNTX-IV indeed form. It is speculated that rHNTX-IV folded naturally in an *in vitro* manner, although the mechanism is unclear.

The stability of the GST-SUMO fused protein seems to be promoted by GST because, in our experience, the His-SUMO tagged rHNTX-IV was prone to aggregate after elution.

Affinity purification as a classical method can yield relative pure products. However, it could be costly for the large-scale production of recombinant peptides. Taking advantage of the hydrophobicity differentiation between the toxin peptides and proteins, purification strategies based on TCA precipitation, or solvent agent (acetonitrile or acetone) precipitation, were economical choices. Our study further supported this fact and suggested that TCA precipitation seems to be more effective than precipitation by organic solvents. This result may be because the cysteine knot toxins, which usually have small sizes and compact structures, are relatively resistant to denaturation from heat, acid/alkali, detergents, and so on [24].

In conclusion, an efficient, simple and cost-effective strategy for rHNTX-IV expression is described in the article and is amenable to large-scale of rHNTX-IV production for further study of activity-structure relationships and pharmaceutical applications. The strategy also provides references for the large-scale production of active peptides with disulfide bonds.
